# Enhanced motor skill acquisition in the non-dominant upper extremity using intermittent theta burst stimulation and transcranial direct current stimulation

**DOI:** 10.3389/fnhum.2014.00451

**Published:** 2014-06-23

**Authors:** Raymond J. Butts, Melissa B. Kolar, Roger D. Newman-Norlund

**Affiliations:** Department of Exercise Science, Arnold School of Public Health, University of South CarolinaColumbia, SC, USA

**Keywords:** TMS, tDCS, brain, motor cortex, learning, Jebsens

## Abstract

Individuals suffering from motor impairments often require physical therapy (PT) to help improve their level of function. Previous investigations suggest that both intermittent theta burst stimulation (iTBS) and bihemispheric transcranial direct current stimulation (tDCS) may increase the speed and extent of motor learning/relearning. The purpose of the current study was to explore the feasibility and effectiveness of a novel, non-invasive brain stimulation approach that combined an iTBS primer, and bihemispheric stimulation coupled with motor training. We hypothesized that individuals exposed to this novel treatment would make greater functional improvements than individuals undergoing sham stimulation when tested immediately following, 24-h, and 7-days post-training. A total of 26 right-handed, healthy young adults were randomly assigned to either a treatment (*n* = 15) or control group (*n* = 12). iTBS (20 trains of 10 pulse triplets each delivered at 80% active motor threshold (AMT) / 50 Hz over 191.84 s) and bihemispheric tDCS (1.0 ma for 20 min) were used as a primer to, and in conjunction with, 20 min of motor training, respectively. Our primary outcome measure was performance on the Jebsen-Taylor Hand Function (JTHF) test. Participants tolerated the combined iTBS/bihemispheric stimulation treatment without complaint. While performance gains in the sham and stimulation group were not significant *immediately* after training, they were nearly significant *24-h* post training (*p* = 0.055), and were significant at *7-days* post training (*p* < 0.05). These results suggest that the combined iTBS/bihemispheric stimulation protocol is both feasible and effective. Future research should examine the mechanistic explanation of this approach as well as the potential of using this approach in clinical populations.

## Introduction

Of the 795,000 people that will suffer from a stroke in the U.S. this year, two thirds will survive and require rehabilitative services (Lloyd-Jones et al., 2009). While new therapeutic techniques have improved functional outcomes by applying the principles of forced use and massed practice, significant stroke-related disabilities often persist following treatment. More than 85% of patients that have suffered a stroke have lasting functional impairments (Wolf et al., 2006) and approximately 50–60% of stroke survivors continue to require functional assistance to complete activities of daily living after completion of intensive physical therapy (PT; Bolognini et al., 2009).

Experiments using fMRI have demonstrated that peripheral motor recovery is accompanied by significant changes within the central nervous system (CNS), suggesting that CNS plasticity plays an important role in the stroke recovery process (Walther et al., 2009). Therefore, techniques capable of modulating cortical plasticity presented in combination with extremity-specific training protocols may provide the key to improved therapeutic techniques and better functional outcomes. Both transcranial magnetic stimulation (TMS) and transcranial direct current stimulation (tDCS) may be capable of initiating such lasting cortical changes.

### Transcranial Magnetic Stimulation (TMS)

Both high-frequency and low-frequency repetitive transcranial magnetic stimulation (rTMS) have demonstrated the ability to induce cortical excitation and inhibition, respectively, that lasts beyond the stimulation time period (Huang et al., 2005, 2008; Huerta and Volpe, 2009). TMS studies incorporating intermittent theta burst stimulation (iTBS) and rTMS have demonstrated >30 min of enhanced motor evoked potentials (MEPs) in target musculature (Huang et al., 2005, 2008; Huerta and Volpe, 2009). Researchers have hypothesized that the beneficial effects of iTBS are related to (i) the creation of theta oscillations within the cortex consistent with those associated with learning and memory in the hippocampus (Huang et al., 2008); and/or (ii) stimulation induced increases in the concentration of brain derived neurotrophic factor (BDNF; Li Voti et al., 2011; Lee et al., 2013).

While only a few studies have explored this possibility and findings are inconsistent, TMS continues to be a promising adjunct to motor training. A recent study comparing 6 h of in-clinic, supervised constraint-induced movement therapy (CIMT) produced equivalent motor skill gains compared to 1 h of in-clinic, supervised CIMT augmented with rTMS and followed by 5 h of unsupervised practice at home (Richards et al., 2006). In a separate study, rTMS was used to stimulate the motor cortex (M1) of stroke patients in conjunction with 10 days of traditional PT, resulting in increased MEPs in target musculature and significantly better scores on clinical and neurophysiological tests (Khedr et al., 2005). Patients undergoing this type of stimulation maintained greater functional independence 10 days after the completion of treatment relative to those receiving sham stimulation, suggesting that semi-permanent cortical changes had occurred (Khedr et al., 2005). Similar gains were also noted by patients suffering from chronic hemiplegic stroke following TMS enhanced hand therapy (Kim et al., 2006). When TMS was applied over the hand region of M1 in the lesioned hemisphere prior to therapy, enhanced motor skill acquisition (Kim et al., 2006) and greater grip-lift kinetics (Ackerley et al., 2010).

### Transcranial Direct Current Stimulation (tDCS)

Transcranial direct current stimulation can be delivered over specific cortical sites to either increase or decrease excitability (Bolognini et al., 2009). tDCS is thought to exert an excitatory effect on cortical neurons by facilitating ion channels, thereby making the resting membrane potential more conducive to depolarization, especially when paired with active movements such as those associated with PT (Fritsch et al., 2010). Like TMS-enhanced rehabilitation, studies incorporating tDCS into motor recovery protocols have yielded promising results. When used to stimulate the contralateral primary motor cortex of healthy right-handed volunteers for 20 min, tDCS enhanced therapy resulted in a 9.4% increase in motor performance of the non-dominant UE compared to a sham control (Boggio et al., 2006). Functional improvements in the dominant upper extremity (UE) following a-tDCS stimulation of the contralateral primary motor cortex were also demonstrated in healthy elderly adults, a population known to suffer from age-related loss of motor function (Hummel et al., 2009). Participants receiving a-tDCS scored 7.98% better on the Jebsen-Taylor hand function (JTHF) test than those that received sham stimulation (Hummel et al., 2009). Importantly, bihemispheric tDCS, in which anodal stimulation is applied over the ipsilateral cortex and cathodal stimulation is applied over the contralateral cortex, has been shown to be even more effective than unihemispheric tDCS in improving function of the non-dominant UE in healthy adults (Vines et al., 2008).

The exact neurobiological mechanisms underlying the capacity of iTBS and bihemispheric tDCS to enhance motor learning are not fully understood. Therefore, it is not straightforward to predict the consequences of combining these two approaches. The purpose of this pilot study was to explore the efficacy and potential of a novel, non-invasive brain stimulation approach to enhancing motor learning, which combined both excitatory iTBS and bihemispheric tDCS. Based on prior positive findings regarding the application of both approaches separately, we predicted that combining both approaches in the current study would have a beneficial effect on motor learning. We selected motor training of the non-dominant UE of healthy college-age students as our performance metric. Multiple studies have demonstrated that the non-dominant UE has relatively less dexterity than the dominant UE, a difference that may be explained by the disproportionate use of the preferred UE and the decreased cortical activation of the non-dominant motor cortex (Özcan et al., 2004; Boggio et al., 2006). Data from TMS further indicates that the non-dominant motor cortex has increased motor thresholds (i.e., greater stimulation is required to elicit movement) and decreased MEPs (i.e., stimulation of equal intensity will elicit smaller MEPs in the non-dominant UE; Boggio et al., 2006; Hamzei et al., 2006).

We hypothesized that motor training of the non-dominant UE, primed with iTBS and administered concurrently with bihemispheric tDCS, would result in better outcomes on the JTHF Test immediately, 24-h, and 7-days post motor training than motor training with placebo stimulation.

## Methods

This was a randomized, double-blind study comparing the functional improvements made by the non-dominant UE per the JTHF. Following consent and screening, participants were randomly assigned to one of two groups: Group 1: iTBS primer followed by bihemispheric stimulation enhanced motor training. Group 2: iTBS sham primer followed by sham bihemispheric stimulation enhanced motor training. In order to establish baseline function, all participants completed a battery of non-dominant UE tasks, which included: JTHF, a computer-based pursuit rotor task, a computer-based reciprocal tapping task, and the Purdue pegboard test ×10 repetitions with the non-dominant UE within 30 h of beginning formal training. In addition, cortical mapping of the non-dominant UE was individually conducted via single-pulse TMS, as described below, and active motor threshold (AMT) was determined.

**Figure 1:**
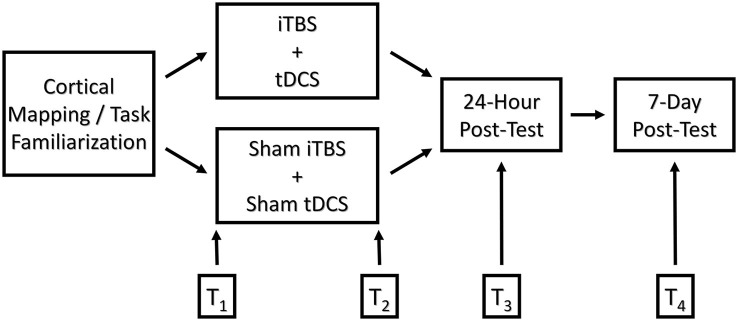
**Graphical depiction of the experimental design comparing functional outcomes of college-age students following motor training augmented with real and sham iTBS/bihemispheric stimulation**. *T*_1_-*T*_4_ represent time points at which motor skill performance was measured: *T*_1_ = pre-test, *T*_2_ = immediate post-test, *T*_3_ = 24-h post-test, and *T*_4_ = 7-days post-test, respectively.

On the day of treatment, participants performed the JTHF ×3 times, and the best two scores were averaged into pre-test measurements. The treatment consisted of a 3-min iTBS primer followed by 20 min of bihemispheric stimulation presented in conjunction with motor training. The control group received sham stimulation. Immediately following, 24-h, and 7-days post-treatment, participants again completed three repetitions of the JTHF, and the best two out of the three scores were averaged. In order to avoid possible circadian effects on performance, the timing of pre and post-tests was kept as uniform as possible (See Figure 1).

### Participant recruitment

Twenty-six healthy participants who met inclusion / exclusion criteria were recruited via word of mouth and flyers placed in and around high traffic areas of the University. Inclusion criteria were as follows: (1) Predominantly right-handed; (2) Age 18–34 years old; (3) Ability to provide informed written or verbal consent. Handedness was verified via performance testing on the JTHF, Purdue pegboard test, computerized pursuit rotor tracking task, and computerized reciprocal tapping task. Exclusion criteria were summarized on TMS, tDCS, and Neurological Screening Forms approved by the Medical University of South Carolina, Department of Neurology. Participants were excluded from the study if they reported the following: cardiac pacemaker, metal on face / scalp, implanted medical pumps / lines, history of stroke / cortical lesion, history of head injury, history of seizures / epilepsy, history of neurosurgery, pregnancy, electrical / magnetic / mechanical implants, history of migraines, report of taking psychiatric medication known to reduce seizure threshold, and any unstable medical condition. Participants were also excluded if they reported a history of dizziness / vertigo, frequent headaches, tremors, strange movements / bizarre behavior, memory problems, double vision, abnormal muscle weakness, unexplained burning / tingling / numbness, sudden change in sleep patterns, extreme or abnormal fatigue, cognitive limitations, and unexplained pain in the hands / feet / face. Prior to enrollment in the study, all subjects read and signed a consent form approved by the University of South Carolina (USC) ethical review board. Participants that satisfied all inclusion / exclusion criteria were randomly assigned to a group (real or sham stimulation). Brain stimulation and data collection was performed at the USC Brain Stimulation Laboratory. All procedures were conducted in accordance with the ethical standards set forth by the Declaration of Helsinki.

### Experimenter blinding

A compatible “jump drive” was encoded with either iTBS (experimental) or sham-iTBS (control) stimulation and assigned to corresponding participants. The same TMS coil was used during both the treatment and control condition. The coil was flipped based on the instructions encoded on each jump drive, making it impossible for the participants or experimenter (Raymond Butts) to determine whether they were receiving real or sham stimulation. In order to ensure participant blinding with regards to bihemispheric stimulation, electrodes were placed on all participants. Participants were informed before-hand that any cutaneous stimulation on the scalp typically decreases with time secondary to desensitization. The tDCS devices were then turned on until participants confirmed that the stimulation could be felt on the scalp. In the Sham group, the tDCS unit was turned off following 30 s of stimulation. A piece of tape was placed over the warning light and selector switches so as to maintain blinding. While the primary investigator responsible for administering stimulation was aware of which group the participants were in, a blinded graduate student unrelated to the study administered all motor assessment testing, thus assuring experimenter blinding.

### Motor training

Motor training focused on the non-dominant UE and consisted of one 20-min session geared toward practicing four primary tasks: the JTHF, a computer-based Fitt’s Reciprocal Tapping Task, a computer-based Pursuit Rotor Tracking Task, and the Purdue Pegboard Test. Of the four tasks, the JTHF was chosen as the primary outcome measure because it provided a short, but relatively broad, measure of hand function. Although the JTHF was designed for patients post-stroke, previous studies have successfully used the test to measure UE, motor performance in healthy young-adults (Boggio et al., 2006). Normative data is readily available for both sexes and various age groups (Hackel et al., 1992). The JTHF is a valid and reliable test that also correlates well with other established standardized assessments such as the Grip strength test, Action Research Arm Test, Nine hold peg test, pinch strength test, and Stroke Impact Scale (Hand domain; Beebe and Lang, 2009) The test was set-up and administered according to a pre-established set of instructions (Jebsen et al., 1969). However, the writing portion of the JTHF was not performed as part of the study due to variation in handwriting and subsequent unavoidable complications standardizing among individuals.

In order to ensure that participants received equal training, participants were instructed to complete all tasks “as quickly and accurately as possible”. In addition, all tasks were performed consecutively in a randomized but predetermined order for the full length of the treatment time. Also, participants performed all tasks ×10 repetitions prior to beginning motor training in order to become familiar with each. Previous research suggests that ×10 trials of the JTHF are sufficient to reach a stable level of performance among participants (Hummel et al., 2005). Moreover, these 10 trials allowed investigators to ensure that participants completed the components of JTHF correctly, uniformly, and to standard.

### Motor cortex mapping and active motor threshold identification

Participants were seated in a comfortable MagVenture treatment chair with the non-dominant hand pronated on a soft surface for comfort. The optimal position for the abductor pollicis brevis (APB) muscle over the scalp (hot spot) was determined using a MagproX100 Magnetic Stimulator, 230 V (MagVenture Inc., Atlanta, GA) and a C-B60 Butterfly Coil. With the handle oriented backward and the coil 45 degrees in the posterolateral direction, single TMS pulses at a predetermined intensity were directed just anterior of the central sulcus and adjusted in 1–2 cm increments until a “hot spot” was identified. A “hot spot” was identified as the location on the scalp able to generate a visual twitch of the APB 3/5 times. The “hot spot” was marked on a cloth MagVenture stimulation cap. In addition to the “hot spot,” the center of the nasal bone, right / left external auditory acoustic meatus and occiput were also marked in order to ensure that the “hot spot” was reliably relocated on the day of treatment. Following the identification of the “hot spot,” participants were then be asked to isometrically grip a dynamometer between the proximal interphalangeal joint of the left D2 and the pad of the left D1 at approximately 20% of maximal voluntary contraction (MVC) while the AMT of the APB was measured. Participants were trained using a hand-held dynamometer to ensure that they were capable of contracting their APB with the appropriate amount of force (20% MVC; Beebe and Lang, 2009). Verbal feedback was provided by the primary investigator during practice to ensure consistency. The AMT was defined as the lowest stimulation intensity able to produce at least 5/10 MEPs greater than or equal to a 200 μV amplitude (above baseline; Huang et al., 2008). All MEPs were visually monitored by the primary experimenter on an appropriately scaled display during measurement of AMT. MEP data were not recorded for offline analysis.

### Transcranial magnetic stimulation procedure

iTBS consisted of three TMS pulses at 50 Hz provided every 200 ms (i.e., at 5 Hz) at 80% AMT of the APB. Ten bursts were grouped and repeated every 10 s for a total of 20 trains. This resulted in a total of 600 pulses per participant. Total stimulation time for the iTBS protocol was 191.84 s. Huang et al.’s (2008) iTBS treatment was directed at each participant’s “hot spot” for APB. TMS stimulation was delivered using a Cool-65 AP Butterfly coil. Depending on the orientation of the coil (i.e., which side was up), it emitted either sham or real magnetic pulses. Therefore, the only difference between the sham and real stimulation conditions was the orientation of the coil. Participants were not queried regarding their knowledge of which condition they were in at any point during the experiment. However, because all participants were naïve to iTBS (and iTBS was done with a different coil than the one used for establishing the motor threshold) it is unlikely that they would have known whether they were in the sham or real stimulation condition.

### Transcranial direct current stimulation procedure

Participants underwent either 20 min of bihemispheric stimulation at 1 mV or sham bihemispheric stimulation. A 20-min duration was chosen so as to be temporally matched with one chargeable unit (the minimum amount of time that a physical therapist can bill a client for). A previous investigation established that 20 min is a safe and effective duration for improving motor performance of the non-dominant UE in healthy, college-age subjects after only one treatment (Boggio et al., 2006). Cortical stimulation (1 mA) was delivered via a pair of saline-soaked surface sponge electrodes (2.5 × 2.5 cm) and connected to a 9-volt battery-driven, constant current stimulator for 20 min (Chattanooga Ionto Iontophoresis System, DJO Global, Vista, California, Salt Lake City, Utah) in conjunction with motor training. The anode was centered on the “hot spot” for the APB. The return electrode (cathode) was placed such that it was directly above a spot the same distance from the midline of the head as the anode, and in the same axial slice, but on the opposite hemisphere. This electrode montage is similar to that used by other experimenters (referred to as bihemispheric stimulation) and is thought to simultaneously stimulate the motor cortex controlling the trained limb and inhibit motor cortex controlling the untrained limb (Lindenberg et al., 2010). Typically, bihemispheric stimulation results in more robust behavioral improvements than unihemispheric (i.e., anode placed over the impaired motor cortex) stimulation protocols (Lindenberg et al., 2013b) although the exact reasons for this are not currently known (Lindenberg et al., 2013a). The only difference between the sham and real stimulation condition was that, 30 s into the sham stimulation condition the experimenter covertly turned the tDCS device off (resulting in a gradual ramp down of the current from 1.0 to 0.0 mV). Prior work using the same setup and approach indicated that participants were unable to tell the difference between sham and real stimulation under these conditions. Although the application of tDCS is not associated with serious negative side effects (i.e., seizure), the combination of an iTBS primer followed by bihemispheric tDCS used in this pilot study was novel. As such, all participants were carefully monitored during administration of both iTBS and subsequent tDCS for any signs of discomfort or any visible motor abnormalities (i.e., twitches, movements of increasing amplitude, etc.).

### Safety aspects of a combined TMS/tDCS protocol

Transcranial direct current stimulation involves the application of weak electric currents (generated by a single 9-volt battery) to change the firing rates of neurons under the scalp. In systematic review articles that have looked at tDCS safety, only mild adverse events such as itchy scalp, fatigue, headache, nausea, and insomnia, were reported (Brunoni et al., 2011). Even after 567 tDCS sessions, which included patients post-stroke, patients with migraine headaches, and patients with tinnitus, investigators reported only an infrequent occurrence of minor adverse events (Poreisz et al., 2007). Like tDCS, TMS may also result in a minor headache or discomfort at the site of stimulation, but an additional risk of TMS is seizures. However, according to the 2009 TMS consensus group, the risk of seizures with repetitive TMS is very low. Out of 3000 studies published within the last 10 years, only 17 have resulted in seizures, 12 of which occurred following parameters that exceeded clinical safety guidelines (Rossi et al., 2009). The current study is in accordance with these safety guidelines.

The adverse events associated with a combined iTBS/ bihemispheric tDCS protocol as described in this study have yet to be delineated. However, studies that have stimulated healthy participants with TMS preconditioned with anodal and cathodal tDCS have not reported any adverse events (Cosentino et al., 2012). Although the second part of our combined approach, tDCS, is not associated with the more serious side effect of seizure, we recognize the safety concerns based on the novelty of the dual approach reported here. As such, all investigators were certified as American Red Cross first responders in accordance with the USC Brain Stimulation Laboratory Policy. Two qualified investigators were present at all times during administration of both iTBS and bihemispheric tDCS. Symptoms and first aid treatment associated with seizures, along with phone numbers for local medical staff, were clearly posted in the lab during all data collection. Participants were carefully monitored during administration of both iTBS and subsequent tDCS for any signs of discomfort or any visible motor abnormalities (i.e., twitches, movements of increasing amplitude, etc.).

### Statistical analysis

We conducted a 2 × 4 repeated measures Analysis of Variance (ANOVA) was performed on total JTHF time (in seconds) to determine the main effect of time (pre-treatment, post-treatment, 24-h post-treatment, and 7-days post-treatment), condition (iTBS/bihemispheric stimulation enhanced motor training and placebo control), and the interaction between time and condition. We conducted three additional targeted *t*-tests to (i) examine differences between the two groups immediately following treatment (DV = pretest score − posttest score) and (2) examine differences between groups at 24 h (DV = pretest score − 24 h posttest score) and examine differences between the groups at 7 days (DV = pretest score − 7 day posttest score). In all cases, we predicted that performance improvements would be greater in the stimulation as compared to the sham groups (hence we used one-tailed *t*-tests).

## Results

All subjects tolerated the combined iTBS/bihemispheric stimulation protocol well, and no adverse reactions related to either treatment were reported. All participants underwent training prior to the treatment to achieve a performance plateau. Of the 27 participants in the study, the data of one student was not considered as part of the analysis due to side effects of prescription medication, including apathy, extreme fatigue, and limited attention. Pre and post-test JTHF scores were consistently >3 standard deviations from the other participants, regardless of group.

**Figure 2:**
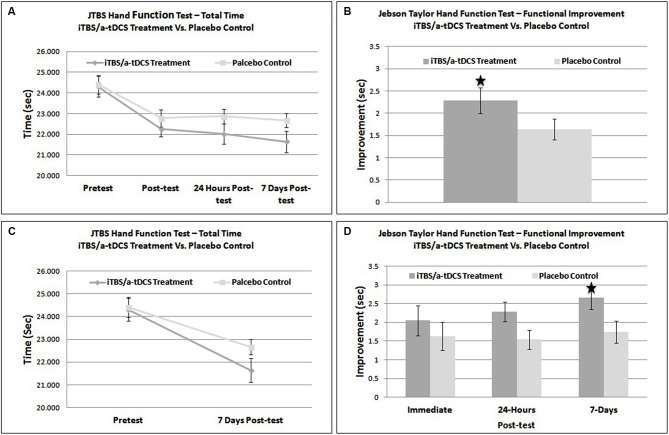
**(A)** Graphical representation of overall functional improvement on the JTHF test. **(B)** Direct comparison of improvement (post-test – pre-test) on the JTHF test in sham and treatment groups. **(C)** Direct comparison of total performance time on the JTHF test in sham and stimulation groups. **(D)** Results of the JTHF test at each time point (immediate post-test, 24-hrs post-test, and 7-days, post-test). The dark gray and light gray line represents the iTBS/bihemispheric stimulation experimental group and placebo-control group, respectively. * = significant effect at *p* < 0.05.

Plots of scaled residuals were created for all JTHF scores using Statistical Analysis SPSS version 22 to ensure homogeneity of variance and normality. A 2 × 4 repeated measures Analysis of Variance (ANOVA) was performed on total JTHF time to determine the main effect of time (pre-treatment, post-treatment, 24-h post-treatment, and 7-days post-treatment), condition (iTBS/bihemispheric stimulation enhanced motor training and placebo control), and the interaction of time and condition. The 2 × 4 repeated measured ANOVA showed a non-significant effect for group (*F*_1,25_ = 0.05, *p* = 0.8322) but a significant effect of time (*F*_2,50_ = 98.05, *p* < 0.0001). The interaction between group and time was also statistically significant (*F*_2,50_ = 5.05, *p* = 0.034) (Figures 2A,B).

A one-tailed between subjects *t*-test comparing *immediate* improvement (pretest − immediate posttest) between the sham and stimulation conditions was not significant, *t*_(24)_ = 0.41, *p* = 0.47. The iTBS/bihemispheric stimulation treatment group outscored the control group by only 1.7% (.414 s). Therefore, the enhanced motor training did not result in greater functional improvements than the sham stimulation. The same statistical test, this time using *intermediate* improvement (pretest score − 24 h posttest score) as the dependent variable was nearly significant, *t*_(24)_ = 2.02, *p* = 0.055 (Figure 2B). When run using *longer-term* improvement (pretest score − 7 day posttest score) as the dependent variable, a similar *t*-test was significant, *t*_(24)_ = 2.13, *p* = 0.04. At 7-days, individuals in the iTBS/bihemispheric stimulation treatment group improved their JTHF score by 11%, outscoring the control group by 3.8% (.92 s) (See Figures 2C,D). Based on a comparison of the improvement scores in the sham and stimulation conditions, Cohen’s d was estimated to be 0.16 immediately following training, 0.82 at 24-h, and 0.86 at 7-days.

## Discussion

The goal of the present study was to explore the feasibility and effectiveness of a novel brain stimulation protocol that used an iTBS primer coupled with bihemispheric stimulation augmented motor training to improve motor learning in the non-dominant UE of healthy young adults. To our knowledge, this is one of the first studies to combine two modalities of brain stimulation in a single training protocol. The first finding of this experiment is that participants in the stimulation group tolerated the combined iTBS/bihemispheric stimulation protocol without complaint. No participant reported any adverse effects at any time during the experiment. While this does not mean that other individuals (perhaps with stroke or other clinical issues) will necessarily tolerate this treatment, it is a positive sign regarding future use of this protocol in clinical populations.

Predicting the effects of stimulation protocols that combine multiple modalities is difficult based on the current state of knowledge regarding iTBS and tDCS. Analysis of the change scores in the sham and treatment groups revealed that there was a significantly greater improvement in the stimulation groups’ motor performance, as compared to performance in the control group. This difference was not present immediately following training, but rather, began to appear at the 24-h time-point and was most robust at the 7-day time-point. While it is unclear what mechanisms supported the observed improvement in the stimulation group, previous literature on the effects of TMS and tDCS on motor learning are consistent with the idea that our protocol exerted its beneficial effects by increasing excitability in the contralateral motor cortex while reducing inhibitory signals (transcolossal inhibition) from the ipsilateral motor cortex (Huang et al., 2008; Bolognini et al., 2009; Huerta and Volpe, 2009; Lindenberg et al., 2010, 2013a,b). This, in turn, may have resulted in greater plastic changes occurring in either the contralateral or ipsilateral primary motor cortex during motor training. While this is a particularly parsimonious explanation for the positive effects of the novel combined treatment, more research is needed in order to further specify the exact nature of both the peripheral and cortical changes associated with this protocol.

Altering the excitability of the motor cortex via non-invasive brain stimulation may help augment traditional motor training and improve functional outcomes. While the present study was conducted on healthy young adults, non-invasive brain stimulation may also improve outcomes of PT used to improve paretic UE function in patients post-stroke. Results of previous investigations have demonstrated a 9.4% improvement in non-dominant UE function per JTHF following motor training primed with a-tDCS compared to sham stimulation (Ackerley et al., 2010). An 8.4% improvement following iTBS/bihemispheric stimulation augmented motor training in the present study is therefore in-line with previous investigations. While a 0.92 s difference (on the JTHF test) between the 7-day improvements scores in the stimulation and control groups appears to be relatively small, the relative difference could be greater in patients who presumably have more room for improvement.

Theoretically, it is possible that combining iTBS and bihemispheric stimulation could have a greater effect on motor skill learning than either of the brain stimulation techniques alone. Critically, this study only compared motor training augmented with iTBS/bihemispheric stimulation with motor training and placebo stimulation. We did not directly compare the effects of our combined iTBS/bihemispheric stimulation to that of iTBS or bihemispheric stimulation alone. Future studies might compare the effects of the combined treatment with each treatment in isolation in order to provide data regarding possible synergistic or antagonistic effects when delivering both TMS and tDCS in close proximity with one another. The current study would have additionally benefitted from inclusion of various physiological measures, such as short-interval intracortical inhibition, and MEP amplitude in the various conditions. Without such physiological data it is difficult to draw conclusions regarding the mechanisms that support the increased performance observed in the experimental group, or to determine if there were any interactions (synergistic or otherwise) between the iTBS protocol’s effects or the effects of the bihemispheric stimulation protocol. Additionally, we recommend more robust studies with larger sample sizes to further investigate the potential of the combined iTBS-bihemispheric stimulation protocol along with the time course of their additive effects. Finally, future studies should also work to correlate functional improvements with reliable measures of cortical excitability and depression.

## Conflict of interest statement

The authors declare that the research was conducted in the absence of any commercial or financial relationships that could be construed as a potential conflict of interest.
